# A Plagiarism Exposed

**Published:** 1893-10

**Authors:** J. M. Thomas

**Affiliations:** Atlanta, Ga.


### A PLAGAIRISM EXPOSED.

*To the Editors of the Southern Medical Record:*

GENTLEMEN:—While looking over the book of the Transactions of the 44th Annual Session of the Medical Association of Georgia, my attention was forcibly directed to the remarkable resemblence between an article, therein contained, by Dr. C. Evans Johnson, editor of the *Journal of Surgery, Gynecology and Obstetrics*, entitled, "Sterility in the Male," and a chapter in Gross' book "Disorders of the Male Sexual Organs." On comparing this article with the chapter on sterility in the second edition of Gross' book, the origin of Dr. C. Evans Johnson's paper was so evident that I deem it but right and proper to call the attention of the profession to this bold plagiarism through the columns of your journal. As will later become evident, it was, however, soon found that several passages which occurred in Dr. C. Evans Johnson's article could not be found in the book just mentioned; this, for a time, greatly puzzled me as the source of the mysterious paragraphs could not be arrived at, but, after a thorough search into the literature of the subject, their origin was fortunately made clear by finding the same paragraphs, often, as in the other case, slightly altered, in Allen's translation of Ultzmann's work on "Genito-Urinary Nemoses." For the sake of convenience I will employ parallel columns, as the order in which the statements of these authors are made, are in most cases changed; these changes, together with the verbal ones, are sometimes of such a radical nature that the original sense is greatly altered, apparently due to a want of a clear understanding of the subject. Below I give literal quotations from Dr. C. Evans Johnson's paper as it occurs in the transactions of the Association, followed by the originals. Occasionally I use italics for the purpose of emphasizing certain words and passages, and to indicate that no mistake has been made in the quotations:

Johnson, p. 114; "Sterility in the Male." Transactions Fourty-fourth Annual Session Georgia Medical Association, 1893.

"A popular error among the laity, and, indeed, among a large proportion of the profession, in all cases of sterile marriages, and more especially when the female is the more delicate of the two, is to lay the fault at her door. Nor is she slow to accept the situation. It has been the custom for her to consult a gynacologist of more or less repute, submit to an examination, and, if deemed necessary, to undergo local and general treatment with the vain hope of correcting this supposed defect, without one thought being given to an examination of the semen of the husband so long as he seemed healthy and was able to copulate and have an ejaculation. But of late years considerable advances have been made in this line by some of the more progressive men in the profession. The truth has gradually become established that ability to perform the sexual act to the satisfaction of both parties does not necessarily imply that the male is imbued with procreative powers.

Sterility in the male does not always have as an accompaniment impotence, as the power to perform the act of coitus is often found unimpaired in cases where the seminal fluid is absolutely sterile. Cases of sterile marriages are often brought to our notice where the husband is found to be potent to a remarkable degree, perhaps, and is able to discharge his conjugal duties perfectly in every respect, but the union remains sterile because of the unfruitfulness of the semen of the male. Indeed, it is a well known fact that so-called impotent men who attempt coitus

Ultzmann, p. 111: "Genito-Urinary Nemoses." Allen's Translation, 1889.

"Our knowledge of the *potentia generandi* in men is of the most recent date. Only a few years ago the opinion was held that men who are able to perform the act of coitus to the satisfaction of both parties, must also possess procreative power. In case of unfruitful marriage, all the blame was usually thrown on the wife. She was subjected, sometimes, to operation, to local treatment and baths, for the most part, however, without attaining the desired result. Only since the male semen has begun to be more closely examined microscopically, and since the morbid processes which lead to sterility in the male have come to be better understood, has the truth gradually become established that *potentia coundi* in the male does not always imply *potentia generandi. Potentia generandi* depends solely upon the procreative power of the semen, while *potentia coundi* signifies the ability to perform the act of coitus, and is often found unimpaired in cases where the semen is absolutely sterile. It is a well-known fact that so-called impotent men, who attempt coitus with flacid penis, are still sometimes able to impregnate the female, if they only possess a normal semen. Accusations of paternity are not rare in this connection. There are also in literature cases of impregnation of virgins with a wholly intact, narrow hymen, where the ejaculation—in order to avoid impregnation—had taken place at the vulva or its neighborhood.

Prof. G. Braun, sometime ago, published some very interesting cases of with a flaccid penis, have been able to impregnate the female where the semen is normal. Occasionally a report of a case will be seen of conception in the vagina where the hymen is found wholly intact, and it will be found that to avoid impregnation the ejaculation has taken place at the vulva. It would seem then that the essential factor in the process of conception is the quality of the semen ejaculated.

"Semen is the mixed product of the secretions of the testes, seminal vesicles vasa deferentia, sinus pocularis, prostrate and Cowper's glands, together with the mucous follicles of the urethra. The thick, whitish secretion from the seminiferous tubes principally consists of seminal cells, of which the spermatozoa are developed.

"The amount discharged at any one time varies with different individuals and at different times in the same individuals ; ordinarily, however, it is between ten and fifteen grammes.

Normal semen has a whitish color resembling very much boiled starch paste, a peculiar characteristic, heavy odor, and, as expressed by Gross, not unlike that of raspings of fresh horn or bone, with an ankaline reaction. Immediately after ejaculation its conthis class. It is, moreover, also a well-known fact that here and there in obstetric clinics girls in labor are met with, in whom the hymen is found entirely intact. The power of procreation depends, for the most part, only upon the quality of the man's semen. As proof of this sterile marriages are often brought to notice, where the husband is remarkably potent and fulfils his conjugal duties perfectly in every particular ; yet the marriage remains sterile, because the semen of the husband is unfruitful." (Here a short paragraph is omitted which has no relation to the present subject.) "Since *potentia generandi*, as already mentioned, depends, for the most part, on the good or bad condition of the semen, first of all should be considered the male semen in health and disease."

The scene now shifts to page 80 of "Disorders of the Male Sexual Organs, by Gross, 1883.

"Semen is the mixed product of the secretions of the testes, vasa deferentia, seminal vesicals, sinus pocularis, prostate, Cowper's glands, and the mucous fallicles of the urethra. The thick, white, pasty secretion of the seminiferous tubes consists mainly of spermatoblasts, or seminal cells, out of which the spermatozoa, or fertilizing elements are developed." * * * *

We return now to Ultzmann's book, page 84.

"The amount discharged at one time is sometimes greater, sometimes smaller, according to the continence of the producer; ordinarily it is between 10 and 15 grammes. Normal semen has a whitish color, resembling boiled starch paste, a peculiar characteristic, heavy odor, and an alkaline reaction. Its consistence immediately after ejaculation is honey-like and ropy; it soon, however, stiffens like gelatine, to become again yet more sistence is ropy and honey-like, it soon, however, stiffens like gelatine, to become again yet more fluid in the course of five or ten minutes. Upon pouring the semen into a test tube and allowing it to settle, it willl be found to separate into two layers of equal bulk. The upper layer is turbid and translucent, containing only a few cells and some detritus, while the lower layer is white, apaque, and consists of spermatozoa and the celular constituents of the semen. It is claimed by Ultzman that from the thickness of the layer containing spermatozoa it is sometimes possible to arrive at a conclusion regarding the generative power of the specimen in question."

"According to Vaquelin, semen consists of 10 per cent. solid matter and 90 per cent. of water. Of solid matter 6 per cent. consists of organic constituents, including the spermatozoa, 3 per cent. of earthy phosphates and 1 per cent. alkalies (chloride of soduum.) It is claimed by Hoppe-Seyler that semen also contains an albuminous substance called spermatin, which, in its reaction, bears resemblance to casein.

"When a specimen of normal semen has been allowed to stand a considerable time, on the second or third day are found, at first only single, later in greater numbers, colorless, transparent, rhomboidal chrystals of phosphoric acid and magnesia, which have been termed spermatic crystals."

fluid in the course of five or ten minutes. The semen, having been poured into a test-tube and allowed to settle, is found, after some hours, to have separated into two layers. In normal semen the two layers are of equal bulk. The lower layer is white, opaque, and consists of the celular constituents of the semen, in normal semen of spermatozoa. The upper layer is turbid and translucent, the microscope showing only a few cells and some detritus. From the thickness of the layer containing spermatozoa, it is sometimes possible to arrive at a conclusion as to the generative power of the semen in question."

We now turn to page 113 of the same book:

"According to Vaquelin, semen consists of ten per cent. solid matter and ninety per cent. water. Of the solid matter six per cent. consists of organic constituents including the spermatozoa, three per cent. of earthy phosphates, and one per cent. of alkalies (chloride of soduum.) According to Hoppe-Seyler, semen contains an albuminous substance called spermatin, which, in its reactions, bears a resemblance to casein.

Continuing, we find on pages 85-86, the following:

"When a normal semen has been allowed to stand a considerable time, on the second or third day are found, at first only single, later in greater numbers, colorless, transparent, rhomboidal crystals. When incompletely crystallized, these crystals appear tapering and rounded at the ends. A. Bottcher considers them albuminoid bodies. Other authors ammonias-magnesic phosphate. I have also examined these crystals, and found that they consist of phosphoric acid and magnesia; ammonia I could not find."

"The quick or tardy appearance of these crystals affords an inference as to the fructifying power of the semen. If a specimen contain manyand living spermatozoa, the crystals appear very late, sometimes not until the third day, because crystallization is impossible in a fluid full of movement. If however, the specimen contain motionless spermatozoa, or none at all, the crystals appear in half an hour."

"The spermatozoa of normal semen consists of an oval or flattened, pear-shaped, shovel-like head, and a long thread-like end which is divided into a middle piece and a tail. The middle piece and tail should be at least ten times the length of the head. The spermatozoa should be present in large numbers, and still show movement at least twelve hours after ejaculation."

"Semen begins to be secreted about the epoch of puberty, and continues to be formed until an advanced age, though the subject is usually rendered sterile from impotence after the seventieth year. Duplay and Dieu, together with Liegeois, have, however, found that old men in the enjoyment of good health are as liable to produce zoosperms as younger men, the latter finding them in thirteen consecutive examinations. Wagner has detected spermatozoa in the fluid emitted by sexagenarians and septenarians. Curling reports them in a man eighty-seven, and Caspar met with them at ninety-six years.

I now refer you to pages 91–92, of the same book :

"The quick or tardy appearance of these crystals affords an inference as to the fructifying power of the semen. That is to say, if a semen contain many and living spermatozoa, the crystals appear very late, sometimes not until the third day, because crystallization is impossible in a fluid full of movement, such as a normal semen containing many living spermatozoa. If, however, the semen contain motionless spermatozoa or none at all, then the spermatic crystals appear in the course of half an hour."

We will now return to page 85:

"The spermatozoa of normal semen consist of an oval or flattened, pear-shaped shovel-like head, and a long thread-like end which is divided into a middle piece and a tail. The middle piece and tail should be at least ten times the length of the head. The spermatozoa should be present in large numbers, and still show movement at least twelve hours after evacuation."

I now refer the reader to pages 84, 85 and 86 of Gross' book:

"Semen begins to be secreted at the epoch of puberty, and continues to be formed until an advanced age, although the sexual power is usually lost after the sixty-fifth* year. * * * Previous to the researches of Duplay in 1852, and of Dieu in 1867, the opinion was very general that the semen of old persons was as infertile as was that of impubic boys, although Wagner has noted the presence of spermatozoa in sexagenarians and septenarians, and Curling and Casper have met with them, respectively, at eighty-seven and ninety-six years. That old men in the enjoyment of good health are as able to produce

*In a later addition the time is placed at the seventieth year.

"Sterility in the male may be classified thus: Azoospermism, or the condition in which unproductive semem is secreted; aspermatism, or where the spermatic fluid is not ejaculated; and mis-emission, or the failure to deposit the seminal fluid in the upper portion of the vaginal canal. In the first variety, we have a condition in which intercouse and ejaculation is normal, but the semen secreted is lacking in the essential anatomical elements — the spermatozoa — which may be absent altogether either because they are not formed or because they are unable to be ejaculated through some obstacle seated in the vasa deferentia, or dead because they cannot exist in the medium in whicq they are suspended. In the second variety, the patient may be wholly capable of copulation, but that act is not concluded with an ejaculation either *because of non-secretion of semen** or because of some impediment to its passage. In the third variety, coitus is possible and fruitful semen may be ejaculated, but because of congenital deficiencies of the urethra, abnormal positions of the meatus or fistulous openings from the urethra, the seminal fluid does nol reach its destination."

"Azoospermism is probably the most frequent of the varieties named. It may be congenital or acquired, *permanent or temporary.** Of the congenital forms we have bilateral anorchidism, or absence of the testes; cryptorchidism, or non-descent of the testicles, bilateral deficiencies of the epididymis and vas deferens. The acquired cases are due to various affections of the testes

*Italics mine.

zoosperms as younger men is shown by the investigations of Liegeois, who discovered them in every examination, thirteen in number, of the fluid emitted by that class of persons."

* * * * * "Sterility includes, first, azoospermism, or the condition in which either no semen whatsoever, or unproductive semen, is secreted; secondly, aspermatism, in which spermatic fluid is not ejaculated; and, thirdly, misemission, or the failure to deposit fertile semen in the upper portion of the vagina. In the first variety intercourse and ejaculation are natural, but the essential anatomical elements are absent or dead, either because they are not formed or are imprisoned behind an obstacle seated in the epididymes or vasa deferentia, or because they are unable to live in the medium in which they are suspended. In the second variety the ability to copulate is unimpaired, but the power to ejaculate is prevented by an impediment situated between the seminal vesicles and the urinary meatus. In the third variety coition and emission are perfect; but fruitful semen fails to reach its proper destination, in consequence of congenital deficiencies of the urethra, or of fistulous openings in that canal resulting from inflammation, or of abnormal positions of the meatus."

On page 87 he states: * * * "The cause of the sterility was azoospermism in thirty-one, and aspermatism in two." * * * * * * * * * "Azoospermism may be due, first, congenital bilateral anorchidism; secondly, to congenital bilateral deficiencies of the epididymis or vas diferens; thirdly, to cryptorchidism; fourthly, to affection of the testes; and to obstruction or obliteration of the *epididymides** or vasa deferentia or both. They may be permanent, as in the first named instance of congenital bilateral anorchidism in congenittal bilateral deficiencies of epididymis and vasa deferens, in complete obliteration of the epididymides and vasa deferentia from gonorrhea and in cryptorchidism. However, in the latter instance, there is yet some doubt as to whether these cases should be classed among those of permanent azoorpermism.

*Italics mine.

"Poland* and Debrou report cases of married cryptorchidis who procreated children."

*Guy's Hosp. Reports, Series 2, Vol. 1.

"Beigel† speaks of the case of a man whose testes were retained in the groins, and whose semen, upon examination, disclosed spermatozoa."

†Virchow's Archiv.

"And temporary absence of the spermatozoa in perfectly healthy subjects may be induced by excessive sexual indulgence, frequent repetition of the sexual act rendering the semen more and more watery, until finally it consists simply of the secretions of the accessory ducts. Leigeois reports the case of a medical student who indulged in *four or five** connections daily for ten consecutive days. Examination of the ejaculated fluid showed total absence of spermatozoa, but upon abstinence for a period of three or four weeks they were found in abundance. Caspar reports an interesting case of this nature, the subject being a vigorous man of sixty years, a naturalist, and father of a large family. He was a

*Italics mine.

fifthly, to obliteration or obstruction of the epididymes or vasa deferentia, and sixthly, to abnormal conditions of the semen. Hence, the affection may be congenital or acquired, absolute or relative."

Gross, on page 90 of his book, says: "On the whole, the evidence in regard to cryptorchids shows that while, as a rule, they are potent, and ejacute a fluid which is devoid of spermatozoa, exceptional instances indicate that they may be fertile."

Again we quote from Gross, page 89: * * * "are the instances recorded by *Poland, Cock, Durham and Debrou, of married cryptorchids who had procreated children."

And still again on page 90:

"Beigel† narrates the case of a man, two-and-twenty years of age, whose testes were situated in the groins, and whose emitted semen disclosed spermatozoa."

We find on page 94, in Gross' book, the following:

"Temporary absence of the spermatozoa may be induced in perfectly healthy men, by exural excesses, and the frequent repetition of the act of coition renders the semen more and more watery and scanty, so that it consists merely of the secretions of the accessory glands. In the case of a medical student, recorded by Liegeois, who indulged in three or four connections dailyfor ten successive days;repeated examinations of the emissions demonstrated the complete absence of spermatozoa, Some months later, after an abstenance of three weeks, they were detected in large numbers. The case of Caspar is so interesting in this respect that it is quoted entire:

very intelligent man and accustomed also to the use of the microscope. The author had interested him in this subject and says :

Here follows the record of this case which is substantially the same in both.

" *With the exception of this last variety*,* the more common are probably those cases of complete obliteration of the tracts of the epididymides and vasa deferentia due to bilateral epididymitis following gonorrhea.

*Italics mine,

"Aspermatism, or that condition in which the patient, whether in coitus or under sexual excitement, is unable to ejaculate semen, is either absolute or relative, permanent or temporary. The absolute and permanent form is somewhat rare, and is either congenital or acquired. Aspermatism may depend upon bilateral anorchidism, obstruction or occlusion of the ejaculatory ducts, absence of sensibility of the nerves supplying the penis, and psychical influences.

We refer again to Gross, pages 91–92:

"By far the most frequent and important of the causes of azoospermism is bilateral obliteration of the epididymis and vas deferens, through which the proper secretion of the testes is confined and is prevented from reaching the vesiculae seminales and the urethra, and the ejaculated fluid is of necessity deprived of spermatozoa. Obliteration of the seminal passages, as Gosselin first pointed out, is usually due to gonorrhœa, when it is, with few exceptions, confined to the epididymes, the vasa deferentia alone being rarely involved."

We quote now from Ultzmann, page 116:

"*Aspermia*—Absence of Semen. By this is understood a condition in which the patient, whether in coitus or other sexual excitement, is enabled to ejaculate semen. Aspermia is either absolute or relative, permanent or temporary. The absolute and permanent form of aspermia is rare. It is either congenital or acquired."

Gross, on page 113 of his book, has the following to say respecting aspermatism.

"Non-emission may be congenital or acquired, and permanent or temporary ; and it may depend, first, upon obstruction of the ejaculatory ducts or the urethra ; secondly, upon deficient excitability of the spinal ejaculatory centre ; thirdly, upon abolished sensibility of the nerves of the penis ; and, fourthly, upon the inhibitory action of the brain over the centre for ejaculation."

"Schmitt and Munroe* report similar cases. * * * * * *

*Boston Med. and Surg. Jour. Feb. 1869.

"From a study of four cases of anorchidism, Godard found that persons in this condition resemble eunuchs mutilated in early life, in that they have no venereal desire, and while they may have, in exceptional cases, erections, they are absolutely sterile."

"More common than the congenital and permanent absence of semen are the acquired forms, which is usually brought about by affections of the prostrate as purulent labar prostatitis, and where both, ejaculatory ducts have become occluded and the glandular tissue of the prostate itself more or less destroyed."

Ejaculation may be prevented also by stricture of small calibre or a tight prepuce.

Here follows the report of two cases from Ultzmann's book. Due credit is given in both cases and no substantial alterations have been made.

Now we refer to Gross, page 114 :

* * "Schmitt* examined a man, thirty-five years of age, who had never an emission either when awake or asleep, although his power to cohabit was unimpaired."

* * "Monroe† describes a similar condition of affairs in a robust man, twenty-eight years of age."

Continuing from the same authority, we find on pages 87–88, of his book, the following :

* * "From a study of four cases, Godard found that persons in this condition resembled eunuchs mutilated early in life. They have no venereal desire, and although they may have, as an exception, erections, they are absolutely impotent and sterile."

Ultzmann says, on pages 117–118, of his book:

"More common than congenital and permanent absence of semen are the acquired forms. The acquired form of permanent aspermia are usually brought about by affections of the prostate. Not infrequently purulent labor prostitis or suppuration of the whole prostrate has preceded. In the acquired form of permanent aspermia, both ejaculatory ducts must have become occluded and the glandular tissue of the prostate itself have been for the most part destroyed."

Gross says, in speaking of organic aspermatism, on page 114, of his book, that it may be due to stricture of that passage (i. e. urethra,) to a tight phimosis, or to induration of the corpora cavernosa.

*Wurzburg Med. Zeitschieft Bd. iii. 1862. p. 361.
†Boston Med. and Surg. Journal, Feb. 21, 1867, p. 62.

In these cases, however, semen is found to ooze out after coitus is finished.

Numerous writers have reported cases of aspermia relieved wholly by circumcission.

" Reliquet has shown that asperma-tism may arise from obstruction of the ejaculation ducts by sympexions or con-cretions composed of spermatozoa, epitheal cells, etc."

These can usually be detected and broken up by the finger in the rectum, and proving as a rule, only a temporary affection.

A case of non-ejaculation of semen is reported by Curling due to an anæsthe-tic condition of the nerves of the glands penis. A cure was affected by an appli-cation of acetum *cantharides** to the part which left it in a very sensitive tive condition. The man subsequently merried and ever afterwards rarely failed to ejaculate during coitus.

Aspermatism from the inhibitory ac-tion of the brain over the ejaculatory center is a common *effection** and is always temporary or relative. Patients will be found who, through disgust or suspicion of infidelity, lack of passion, etc., are unable to complete sexual intercourse with their wives or some

*Italics mine.

And on page 119 * * * When the obstacle, on the other hand, is sit-uated at the external orifice, the semen will dribble away with the subsidence of the erection.

After mentioning cases of the same kind in his own experience, he says on page 121, "In a similar instance Blackwood circumcised the patient and relieved hls trouble."

Continuing, we find on page 117, the following :

"Aspermatism may arise, as Reli-quet first pointed out, from *obstruction of the ejaculatory canals by sympexions*, or concretions composed of spermato-zoa, concrete mucous, epithedial cells and refracting granules, and formed in the seminal vesicles." And on page 129, "When the ejaculatory ducts ore ob-structed, the plan proposed and suc-cessfully practiced by Reliquet in two cases is to be recommended. A sound having been introduced into the blad-der, the circumscribed swelling is emp-tied by counterpressure with the fin-ger in the rectum."

" On page 125 of the same book the following occurs:

* * * * Concluding that the trouble arose from a want of excita-bility in the nerves of the gland of the penis, Curling applied the acetum can-tharidis, which left the part in a very sensitive condition ; and the man sub-sequently married, and seldom failed to finish intercourse in the normal manner."

And again on page 127:

"Other men through disgust, sus-picion of infidelity, or loss of passion, are unable to complete sexual congress with their wives, although they suc-ceed perfectly with other women. Hense, aspermatism from inhibitory ac-tion of the brain over the center for ejaculation is tempoiary or relative, particular woman, although they succeed with others.

Relative aspermia is especially rare. Here we have a condition in which the semen is not ejaculated during coitus, and yet it is emitted during sleep, and while the semen may be rich in spermatozoa, sterility results in that it fails to be discharged into the genital canal.

emission being possible under some circumstances and impossible under others ; and it is altogether independent of organic lesions."

To Ultzmann I will once again refer you, pages 120–121:

"Relative aspermia, it must be admitted, is especially rare. Here we have to do with cases in which the semen can never be produced in coitus, even when, with penis erect, the act is prolonged until exhaustion. As soon, however, as the patient has fallen asleep, he has an emission."

Dr. C. Evans Johnson here again refers to mis-emissions, making substantially the same statements respecting it as in his classification, already referred to ; and he further states, that this condition does not necessarily produce sterility as in exceptional cases, where the semen has been deposited, through a fistulous opening, at the vulva, conception has taken place. The latter statement could not be verified from either of the editions of Gross or Ultzmann to which I had access, but it may occur in one of the later editions of these works, which I am unable to obtain on account of the lack of time. After an unimportant digression Dr. C. Evans Johnson says that plastic operations can be practiced in most of these cases of malformation, and the patient rendered capable of procreation. Just where this opinion originated I am unable to say as both of the authorities for whom he evinces such extraordinary fondness seem on the whole to be inclined to an opposite view. Dr. C. Evans Johnson now closes his paper with the observation that "that there are some points in connection with this subject which might be referred to, but the object of this paper is simply to direct a channel of thought and investigation along this line, which will naturally result in much good." Here again I must confess myself at fault as no such passage can be anywhere found in the literature of this subject at my command. The only thing discovered in this paper which is distinctly original is the unheard of audacity of the author in attempting to palm off as original this glaring fraud on an assembly of educated men.

J. M. THOMAS, M. D.,

Atlanta, Ga.

Sept. 23d, 1893.

